# Neuroinflammation and neurodegeneration following traumatic brain injuries

**DOI:** 10.1007/s12565-024-00778-2

**Published:** 2024-05-13

**Authors:** Matthew Boulton, Ali Al-Rubaie

**Affiliations:** School of Health Sciences, Swinburne University of Technology, Hawthorn, VIC 3122 Australia

**Keywords:** Traumatic brain injury, Neurodegeneration, Reactive astrocytes, Neuroinflammation

## Abstract

Traumatic brain injuries (TBI) commonly occur following head trauma. TBI may result in short- and long-term complications which may lead to neurodegenerative consequences, including cognitive impairment post-TBI. When investigating the neurodegeneration following TBI, studies have highlighted the role reactive astrocytes have in the neuroinflammation and degeneration process. This review showcases a variety of markers that show reactive astrocyte presence under pathological conditions, including glial fibrillary acidic protein (GFAP), Crystallin Alpha-B (CRYA-B), Complement Component 3 (C3) and S100A10. Astrocyte activation may lead to white-matter inflammation, expressed as white-matter hyperintensities. Other white-matter changes in the brain following TBI include increased cortical thickness in the white matter. This review addresses the gaps in the literature regarding post-mortem human studies focussing on reactive astrocytes, alongside the potential uses of these proteins as markers in the future studies that investigate the proportions of astrocytes in the post-TBI brain has been discussed. This research may benefit future studies that focus on the role reactive astrocytes play in the post-TBI brain and may assist clinicians in managing patients who have suffered TBI.

## Introduction

Traumatic brain injuries (TBI) occur in up to 70 million individuals per year (Dewan et al. 2019), and may cause many debilitating consequences. The brain is commonly injured following fractures to the facial region due to the thinness of many of the bones in this area (Kühnel and Reichert 2015; Murray et al. 2017). The most common causes of facial fractures include motor vehicle accidents, assaults and falling injuries (Kühnel and Reichert 2015; Murray et al. 2017; Povolotskiy et al. 2019; Fernandes et al. 2022; Grill et al. 2022), with recent studies showing that from 30 to 55% of facial fractures had resulted in a TBI, with a significantly larger number of males sustaining these facial fractures compared to females (Rajandram et al. 2014; Fernandes et al. 2022; Tsur et al. 2023). When bones in the facial region are fractured, the common areas of the brain to sustain damage are the temporal and frontal lobes (Yang et al. 2021; Tsur et al. 2023).

TBI in the frontal lobes are associated with a variety of complications ranging from short to long-term, with short-term complications including cerebrospinal fluid (CSF) leaks when the skull base is damaged, increased intracranial pressure and risk of haemorrhage (Gatzinsky et al. 2020), and death (Tavarez et al. 2012; Shah et al. 2017; Nasi et al. 2018; Burns et al. 2019; Varshneya et al. 2019; Adatia et al. 2021; Choi and Kim 2022; Pellot and De Jesus 2023). Long-term side effects of TBI may include cognitive impairments (Faulkner et al. 2020; Sullivan et al. 2020; Rostowsky and Irimia 2021; Cao et al. 2022; Zhang et al. 2022), alongside a growing base of literature discussing the impact TBI may have on the development of neurodegenerative diseases such as Alzheimer’s Disease (AD), Parkinson’s, and Amyotrophic Lateral Sclerosis (Gu et al. 2022). The link between AD and certain TBI conditions is much more clearly defined, with Chronic Traumatic Encephalopathy and severe TBI having a large amount of literature showing links with AD pathology and neurodegeneration (Washington et al. 2016; Shao et al. 2022). However, for single mild TBI (mTBI) the link is yet to be completely established, with one case–control study with a sample of 65 finding no links between mTBI and dementia or AD risk (Tsolaki et al. 1997), alongside a more recent 12-year follow-up study with 4144 participants finding no links between TBI with loss of consciousness and dementia or AD (Grasset et al. 2023). However, several recent reviews have concluded there may be links between mTBI and neurodegenerative pathology, with mTBI resulting in AD pathology, including increased amyloid-beta plaques, neurofibrillary tangles, Tau and Lewy pathology (Washington et al. 2016; Wilson et al. 2017; Ramos-Cejudo et al. 2018).

Severe TBI commonly results in neuroinflammation, which activates a variety of immune cells in the brain, including microglia, oligodendrocytes and astrocytes (Nikam et al. 2021). Microglia and astrocytes upregulate and downregulate a variety of proteins to perform various functions (Yuan and Wu 2022). Under pathological conditions, they undergo a shift in morphology and gene expression regulation patterns to perform neuroprotective and neuroinflammatory function in the brain, including the formation of a glial scar to section off injuries, and repair of synapses and the blood–brain barrier (Yuan and Wu 2022).

Recent studies have uncovered different types of reactive astrocytes that are activated following TBI and in neurodegenerative diseases, studied as neurotoxic astrocytes and neuroprotective astrocytes (Liddelow et al. 2017). Astrocytes are initially resting, however, transition into reactive astrocytes following a pathological event; Glial fibrillary acidic protein (GFAP) gene expression is higher in reactive astrocytes, and is commonly used as a marker for reactive astrocytes (Yuan and Wu 2022). Reactive astrocytes upregulate specific proteins to increase or decrease inflammation; neurotoxic astrocytes that increase inflammation have been linked to a compound called Complement Component 3 (C3) which causes apoptosis in surrounding cells (Clark et al. 2019), while other compounds such as Crystallin Alpha-B and S100A100 are neuroprotective in nature and have been linked to neuroprotective astrocytes (King et al. 2020; Hou et al. 2022). Whilst astrocytes themselves may not be visible using neuroimaging techniques, the accompanying neuroinflammation can be seen using a variety of imaging techniques.

Radiographic studies have investigated the changes in the frontal white matter following a TBI using Magnetic Resonance Imaging (MRI) and found hyperintensities in the white matter following TBI, which is associated with impaired cognitive abilities (Zhang et al. 2022). White-matter hyperintensities (WMHs) reveal areas of higher blood flow and are common under pathological conditions due to neuroinflammation (Zhang et al. 2022). Other changes in cognitive abilities which may result from damaged white matter of the brain include acute cognitive impairment and attention deficits in young adults (Cao et al. 2022).

Studies on the brain using Angio-Computed Tomography (Angio-CT) have found a variety of cerebrovascular injuries, including cerebral contusions, cerebral venous sinus injuries and cerebral haemorrhages (Haddad et al. 2022; Chtara et al. 2023). These cerebrovascular injuries have also been discussed to contribute to AD pathology and neurodegeneration in the brain following the TBI (Ramos-Cejudo et al. 2018). Cerebrovascular consequences and reactive astrocytes may all play their own part in the neurodegeneration of the brain and complications which follow TBI, and are all of importance when considering treatments (Zhou et al. 2020).

This review aims to identify the gaps in the current literature regarding TBI effect on the immunohistochemical profile of the brain, alongside establishing a link between TBI, neuroinflammation, reactive astrocyte activation and changes in white matter. Furthermore, future areas of research are discussed regarding reactive astrocytes and areas which are yet to be investigated regarding TBI, neuroinflammation and astrocytes.

## Traumatic brain injuries and head fractures

### Aetiology and specific areas

TBI commonly result from fractures in the neurocranium and frequently occurs following blunt-force trauma to the head (Kühnel and Reichert 2015). Maxillofacial fractures are one of the most common consequences of blunt-force trauma to the head (Kühnel and Reichert 2015). Approximately 30–55% of maxillofacial fractures will also result in TBI (Rajandram et al. 2014; Fernandes et al. 2022; Tsur et al. 2023). The most common causes of TBI are motor vehicle accidents, however, other notable causes include falls, assault, sport-related injuries, industry-related injuries and pedestrian injuries (Rajandram et al. 2014; You et al. 2018). Researchers have also investigated how the severity of facial fractures was related to TBI and found that as the severity of the facial fractures increased, so did the severity of TBI and less favourable TBI outcomes 3 months following TBI (You et al. 2018).

Whilst a variety of bones are in the maxillofacial region, one bone of interest that has been discussed in previous literature is the ethmoid bone, which forms the roof of the nasal cavity with the cribriform plate portion (Kühnel and Reichert 2015; Murray et al. 2017). These fractures which are commonly associated with TBI may have a variety of both short and long-term complications which are of interest to clinicians managing their patients.

## Complications of TBI

### Short-term complications

TBI may be associated with various short-term complications depending on the severity of the fracture and the immune response, such as CSF leaks resulting from a damaged meningeal layer in the skull, and may also result in cerebral haemorrhaging (Shah et al. 2017). For both of these complications increased intracranial pressure may occur, which can cause headaches, blurred vision, stroke and death (Shah et al. 2017). As previously mentioned, depending on the severity of the brain injuries, this may lead to an immediate immune response driven by astrocytes and other glial cells in the brain (Clark et al. 2019). This response occurs as a result of complicated fractures, such as frontal nasal-sinus fractures, which may contribute to CSF leaks and transmission of pathogens into the neurocranium through the nasal cavity (Zele and Dewaele 2016; Alhusain and Aladwani 2019; Rotter et al. 2020). Such pathogens may result in pneumocephalus, a condition where there is increased air in the neurocranium, which commonly occurs in the frontal region following nasal injuries (Yoneoka et al. 2020). When the frontal lobe is damaged due to a TBI, complications may include cerebral abscesses in the frontal lobe (Chtara et al. 2023), pulmonary embolism in the pulmonary arteries (Haddad et al. 2022), or seizures (Son Nguyen et al. 2016), alongside psychological symptoms such as loss of consciousness and confusion which may last from short to long term (Son Nguyen et al. 2016; Thuy-My et al. 2019).

### Long-term complications

Studies have discussed the long-term impact of TBI on the brain, and generally, the symptoms differ based on a variety of factors, including the severity of the injury and the immune response following the incident, which both may play a role in the potential long-term neurodegenerative changes in the brain (Rostowsky and Irimia 2021).

Long-term neurological complications following a TBI have been studied, and long-lasting cognitive impairments such as attention problems post-TBI have been found (Cao et al. 2022), alongside slowed information processing speeds (Rostowsky and Irimia 2021; Zhang et al. 2022). Studies have also found persisting intelligence impairments following TBI in both adults and children, with more severe TBI resulting in more severe cognitive impairments (Königs et al. 2016; Ko et al. 2022). Some studies have tested hyperbaric oxygen treatment on IQ changes and cognitive symptoms following TBI and discovered significant improvements in IQ, memory motor speed and attention (Harch et al. 2017). Furthermore, a variety of larger randomised studies have investigated this relationship and found cognitive and symptomatic improvements following the application of hyperbaric oxygen treatment (Harch 2022). Other researchers have also investigated oestrogen as a potential treatment to reduce TBI-induced inflammation in the brain, specifically finding that oestrogen treatment inhibited neuroinflammation by preventing neurotoxic astrocyte pathways in the brain, however further research is required to deduce the neuroprotective effects that oestrogen may have on the brain and glial cells (Wang et al. 2021).

TBI may result in several further long-term complications to brain health and overall wellbeing (Wilson et al. 2017), with a variety of studies suggesting that neurodegeneration and AD-like pathology resulting from a severe TBI are quite common (Giunta et al. 2012; Heppner et al. 2015; Washington et al. 2016; Wilson et al. 2017; Ramos-Cejudo et al. 2018; Agrawal et al. 2022). Studies have found links between TBI and amyloid-beta plaques, neurofibrillary tangles, Tau and Lewy body pathology (Washington et al. 2016; Wilson et al. 2017; Agrawal et al. 2022). Furthermore, some studies specifically found loss of consciousness (LOC) post-TBI to be a determining factor for neurodegeneration, specifically finding TBI with LOC was associated with increased amyloid-β load (Agrawal et al. 2022) and hippocampal pTau (Postupna et al. 2021). Another study investigated how acute cognitive impairment following TBI may be linked with neurodegeneration, and found that as a degree of cognitive impairment post-TBI increases, so does the likelihood of neurodegenerative brain atrophy (Rostowsky and Irimia 2021). Whilst the specific factors that cause neurodegeneration following TBI are still under debate, understanding the functional changes that occur in the brain after a TBI may provide knowledge on how to best treat the neurodegenerative effects on the brain.

## Immunohistochemical changes in the brain following TBI

### Role of microglial cells and astrocytes post-TBI

The immune response following a TBI has been investigated with a focus on the microglial changes in a post-TBI brain (Clark et al. 2019), with studies discussing two types of microglial cells which have been labelled as M1 and M2 (Clark et al. 2019; Guo et al. 2022). M1 microglia have been reported to increase inflammation and are neurotoxic, whilst M2 microglia are considered anti-inflammatory and neuroprotective (Guo et al. 2022). These categorisations however have been disused by a variety of researchers, as they do not reflect the complexity of gene expression upregulation that microglia facilitate (Escartin et al. 2021). Whilst microglia perform a variety of functions in the brain following TBI, importantly they activate astrocytes in response to inflammation and pathology, which may drastically alter the immune response that occurs in the brain (Liddelow et al. 2017; Xu et al. 2019; Guo et al. 2022).

The response of astrocytes has similarly been categorised into A1 reactive astrocytes which promote pro-inflammatory responses, are neurotoxic and release a variety of chemicals that increase inflammation in the brain, while A2 reactive astrocytes promote anti-inflammatory responses and elicit neuroprotective effects on the brain (Liddelow et al. 2017; Fan and Huo 2021). However, similar to microglia, this categorisation system may not account for the diverse phenotypes that astrocytes possess, with a variety of upregulation patterns which does not conform to only two phenotypes (Escartin et al. 2021). In this paper and similar to previous studies, reactive astrocytes which under pathological conditions such as TBI or AD, upregulate inflammatory gene expressions will be called neurotoxic astrocytes, and those which upregulate anti-inflammatory gene expressions will be called neuroprotective astrocytes (Liddelow et al. 2017; Zhou et al. 2020).

Many authors have discussed these reactive astrocytes and their connexions with AD. A recent review suggested astrocytes form an important role in synapse recovery and loss following a TBI; releasing proteins such as Hevin which may assist in circuit re-organisation following TBI, and enzymes such as matrix metalloproteinase 3 which breaks down extracellular matrixes and inhibits synapse recovery (Jamjoom et al. 2021). They suggested that reactive astrocytes may be associated with the spread of tau through the brain via trans-synaptic transmission, through the systematic transfer of tau pathology throughout the brain via impaired synapses (Jamjoom et al. 2021). This suggests that the role of reactive astrocytes in breaking down synapses via the upregulation of Matrix metalloproteinase 3 and other pro-inflammatory proteins could assist in the spread of tau, which could increase the likelihood of AD pathology (Jamjoom et al. 2021). These upregulation patterns suggest that astrocytes may play a dual role in neuroinflammation and neurodegeneration following TBI, regulating both the inflammatory and anti-inflammatory responses (Liddelow et al. 2017).

Some studies have found increases in neurotoxic astrocytes in individuals with AD (Liddelow et al. 2017), and similarly have found increases in neurotoxic astrocytes in individuals who have suffered from TBI (Clark et al. 2019). Other studies have discussed a more complex relationship between neurotoxic and neuroprotective astrocytes during AD, due to differing densities of these reactive astrocytes’ gene expressions in different regions of the AD brain (King et al. 2020). Their study found that following a TBI, there were increased neurotoxic astrocytes in the upper frontal cortex, and increased neuroprotective astrocytes in the frontal upper and lower cortexes, alongside the white matter of the brain when compared with controls (King et al. 2020). This shows that the distribution of reactive astrocytes is complex, and further research into the distributional activation of reactive astrocytes in the brain following pathology is required (King et al. 2020). Investigating the variety of genes which may be upregulated by reactive astrocytes under pathological conditions such as AD or post-TBI could provide additional insight into the localised functions of astrocytes following trauma and may advance our knowledge of the complex gene upregulation patterns of reactive astrocytes following trauma to the brain.

### Gene expression changes resulting from TBI

Following TBI, (GFAP is commonly released by glial cells (Becerra-Hernández et al. 2022). GFAP is an intermediate filament protein that is released in higher amounts under pathological conditions and has been discussed to assist in the proliferation of astrocytes from resting to reactive (Yuan and Wu 2022), and thus GFAP is commonly used in studies as a marker for reactive astrocytes (Clark et al. 2019; Escartin et al. 2021; Wang et al. 2021; Becerra-Hernández et al. 2022). A list including many gene expressions that are changed following TBI and under neurodegenerative conditions can be seen in Table 1.

**Table 1:** List of gene expression changes by astrocytes under pathological conditions

Gene list	Expression information	Function in brain	Species	Additional information	References
Aquaporin 4 (AQP4)	High expression in reactive astrocytes post-injury	Water channel membrane protein	Murine	Promotes BBB integrity	Zhang et al. 2019)
Glial fibrillary acidic protein (GFAP)	High expression in reactive astrocytes	Intermediate filament	Human, Murine	Biomarker of neurotrauma	Liddelow et al. 2017)
Nuclear kappa factor B (NF-κB)	High in AD compared to control	Transcription factor	Human, Murine	Regulates neuroinflammation	Lawrence et al. 2023; Lian et al. 2015)
Complement component 3 (C3)	High in AD compared to control	Complement factor	Human, Murine	Promotes a variety of inflammatory responses	King et al. 2020; Lian et al. 2015; Stym-Popper et al. 2023)
Aldolase-C (ALDOC)	Secreted by damaged astrocytes	Glycolytic enzyme	Human	Fluid biomarker of neurotrauma	Halford et al. 2017)
Fatty acid-binding protein 7 (FABP7/BLBP)	Secreted by damaged astrocytes	Lipid transport	Human	Fluid biomarker of neurotrauma	Halford et al. 2017)
Crystallin alpha-B (CRYA-B)	Increased expression in contused cortical tissue	Molecular chaperone	Human	Reduces protein aggregation. Inhibits apoptosis through the caspase pathway	Becerra-Hernández et al. 2022; Kuipers et al. 2017)
Nuclear factor of activated T-cells (NFAT)	Found in Alzheimer’s and post-TBI	Transcription factor	Murine	Inflammatory effects, disrupt synaptic remodelling in hippocampus	Furman et al. 2016)
S100A10	High in AD compared to control	Calcium binding protein	Human	Suggested to promote neuroprotective pathways	King et al. 2020)
S100β	High in brains following trauma	Calcium binding protein	Human, Murine	Fluid biomarker of injury	Baecker et al. 2020; Michetti et al. 2019)
STAT 3	May also be expressed in neurons and other cells	Transcription factor	Murine	Promotes synaptic plasticity	Tyzack et al. 2014)
Interferon gamma (IFN-γ)	Expressed by astrocytes following EAE	Cytokine	Murine	Regulates inflammation and limits demyelination	Hindinger et al. 2012)
Synemin	Released by reactive astrocytes in response to TBI	Intermediate filament	Murine	Regulates structural integrity of glial cells	Jing et al. 2007)
Tumour necrosis factor (TNFα)	Higher in post-TBI (between 24-168 h)	Signalling molecule	Murine	May assist glial scar formation The major regulator of inflammation	Chen et al. 2020)
Bone morphogenetic protein (BMP)	High in reactive astrocytes in spinal cord injury	Signalling molecule	Murine	Regulates glial cell differentiation May assist glial scar formation	Wang et al. 2011)
Nestin	Released by reactive astrocytes in response to TBI	Intermediate filament	Human, Murine	Released by both astrocytes and progenitor cells May assist in glial scar formation	Moreels et al. 2008)
Hevin (SPARCL1)	High post-TBI	Synaptogenic protein	Murine	Reported to promote synaptic recovery	Jamjoom et al. 1878; Furman et al. 2016)
Matrix metalloproteinase 3 (MMP3)	Expressed by astrocytes following TBI	Enzyme (Can digest various extracellular components)	Murine	Breaks down extracellular matrix, inhibiting synapse recovery post-TBI	Jamjoom et al. 1878; Falo et al. 2006)
Chondroitin sulfate proteoglycans (CSPG)	High in brain post-TBI	Extracellular matrix component	Murine	Assist in forming glial scar	Harris et al. 2009)
Transforming growth factor β (TGF-β)	Expressed by astrocytes under pathological conditions	Neurotrophic growth factor	Murine	May trigger glial scar formation, assist in synaptic recovery	Xu et al. 2019; Diniz et al. 2012)
Basic fibroblast growth factor (bFGF)	Expressed by astrocytes following TBI	Neurotrophic growth factor	Murine	Improves BBB integrity	Wang et al. 2016)

*AD* Alzheimer’s disease, *TBI* traumatic brain injury, *BBB* blood–brain barrier, *hrs* hours, *EAE* experimental autoimmune encephalitis

### Neurotoxic reactive astrocytes gene expression

Studies have focussed on the secretions of neurotoxic astrocytes and their implications following a TBI and during AD, such as nuclear factor Kappa-B (NF-κB), a protein that is activated following damage to the central nervous system and promotes a variety of pro-inflammatory pathways in the brain (Lawrence et al. 2023). Some researchers have suggested that NF-κB may be upregulated by reactive astrocytes (Kang and Hébert 2011); activating the NF-κB pathway which causes neuroinflammation via the increased transcription of C3 (Lian et al. 2015).

C3 is a protein that is part of the complement system and elicits apoptosis in affected cells (Clark et al. 2019). Researchers have investigated the relationship between Amyloid-β, the NF-κB pathway and C3 production using human control samples with post-mortem AD brains and found higher NF-κB and C3 following exposure to Amyloid-β (Lian et al. 2015). Using lab mice to investigate this further, the researchers concluded that after exposure to Aβ, astrocytes upregulated NF-κB and C3 which caused further increases in Amyloid-β deposits (Lian et al. 2015). Post-mortem human studies have investigated the amount of C3 in the brain of individuals with AD and found 60% of GFAP-positive astrocytes in the pre-frontal cortex of individuals with AD were expressing C3 as well (Liddelow et al. 2017). This fits with previous research regarding GFAP as a marker for reactive astrocytes (Clark et al. 2019; Escartin et al. 2021; Wang et al. 2021; Becerra-Hernández et al. 2022), and signifies that both neurotoxic and neuroprotective astrocytes have their roles in neuroinflammation as previously reported (King et al. 2020). Other studies have supported these findings of increased amounts of C3 in the brains of AD patients, with one study comparing the distributions of neurotoxic and neuroprotective gene expressions in AD patients with reference to controls and found significantly higher C3 in the pre-frontal cortex (King et al. 2020). Studies have also investigated the amount of C3 in the brains of rats following a TBI using a sample of 5 controls with sham injury, and 5 experimental rats with lateral fluid percussion injury (Clark et al. 2019). Their study found increases in the amount of C3 and GFAP in the injured cortex of the brain (Clark et al. 2019). This suggests that astrocytes in the injured cortex following TBI show similar neurotoxic patterns to AD, however, the current literature is yet to investigate the proportion of reactive astrocytes and C3 expression in the post-mortem brain of humans following a TBI.

### Neuroprotective reactive astrocyte gene expression

S100A10 is a calcium-binding protein that promotes neuronal survival and growth (Arranz and Strooper 2019) and assists in intracellular neurotransmitter systems (Rescher and Gerke 2008). Various studies have found that S100A10 is upregulated by neuroprotective astrocytes due to its neuroprotective functions, and it is commonly used as a marker to determine quantities of astrocytes upregulating anti-inflammatory gene expressions (Liddelow et al. 2017; Arranz and Strooper 2019; King et al. 2020). A recent study that examined the distribution of neuroprotective astrocytes in the human brain by immunostaining for S100A10 found low immunopositivity in their control cases for both the white matter and the cortex (King et al. 2020). However, S100A10 expression was increased in the white matter in AD compared to controls, which led researchers to believe that neuroprotective astrocytes were also activated in these areas for individuals with AD (King et al. 2020). Whilst research has investigated S100A10 under neurodegenerative conditions such as AD, we were unable to find any studies investigating the release of S100A10 by reactive astrocytes following TBI. Due to the prior use of S100A10 as a marker for neuroprotective astrocytes (Liddelow et al. 2017; Arranz and Strooper 2019; King et al. 2020), future studies could investigate this protein as a marker for neuroprotective astrocytes following TBI.

Another protein that is upregulated by neuroprotective reactive astrocytes is CRYA-B, which assists in maintaining homeostasis in the cell, alongside protecting the cell from oxidative stress, promoting cell survival pathways, and preventing apoptosis via inhibition of caspase activity (Kuipers et al. 2017; Becerra-Hernández et al. 2022). CRYA-B has been suggested to exhibit neuroprotective effects on the brain following trauma (Shao et al. 2013; Guo et al. 2019; Saglam et al. 2021), and thus has been suggested to be upregulated by neuroprotective reactive astrocytes (Hou et al. 2022) and may be useful for the identification of neuroprotective reactive astrocytes.

### Changes in neuronal cells following TBI

One recent study found that following a TBI, CRYA-B and GFAP were overexpressed in contused tissue which was collected from the frontal and temporal lobes (Becerra-Hernández et al. 2022), with the researchers suggesting this as a marker for reactive astrogliosis, and neuroprotective astrocyte activation in contused tissue. Another study investigated cortical and white-matter changes in the brain following semi-recent severe TBI and found “ballooned” neurons in the cortex and white matter which also expressed CRYA-B, which likely signified the presence of CRYA-B upregulating neuroprotective astrocytes in these damaged neurons following severe TBI (Michaud et al. 2023).

Interestingly, a recent study investigated the morphology of neurotoxic and neuroprotective astrocytes and found neurotoxic astrocytes which express C3 exhibited elongated dendrites, however, neuroprotective astrocytes which express S100A10 showed fewer dendrites and increased cell hypertrophy (Zong et al. 2020; Fan and Huo 2021). This morphological finding of neuroprotective astrocytes exhibiting hypertrophy may be supported by the previous study which found “ballooned” neurons in the presence of CRYA-B, an upregulated protein via neuroprotective astrocytes (Zong et al. 2020; Fan and Huo 2021; Hou et al. 2022; Michaud et al. 2023). Perhaps the neuroprotective astrocytes exhibit effects on the surrounding neurons to change their structure and size, and CRYA-B may perform a role in these changes (Becerra-Hernández et al. 2022). These “ballooned” neurons may be related to MRI studies which have found increases in cortical thickness in the white matter following TBI (Mazaharally et al. 2022), however additional studies that use both histology and neuroimaging are required to verify any relationships.

### Diagnostic methods and neuronal cell inflammation following TBI

**Fig. 1:**
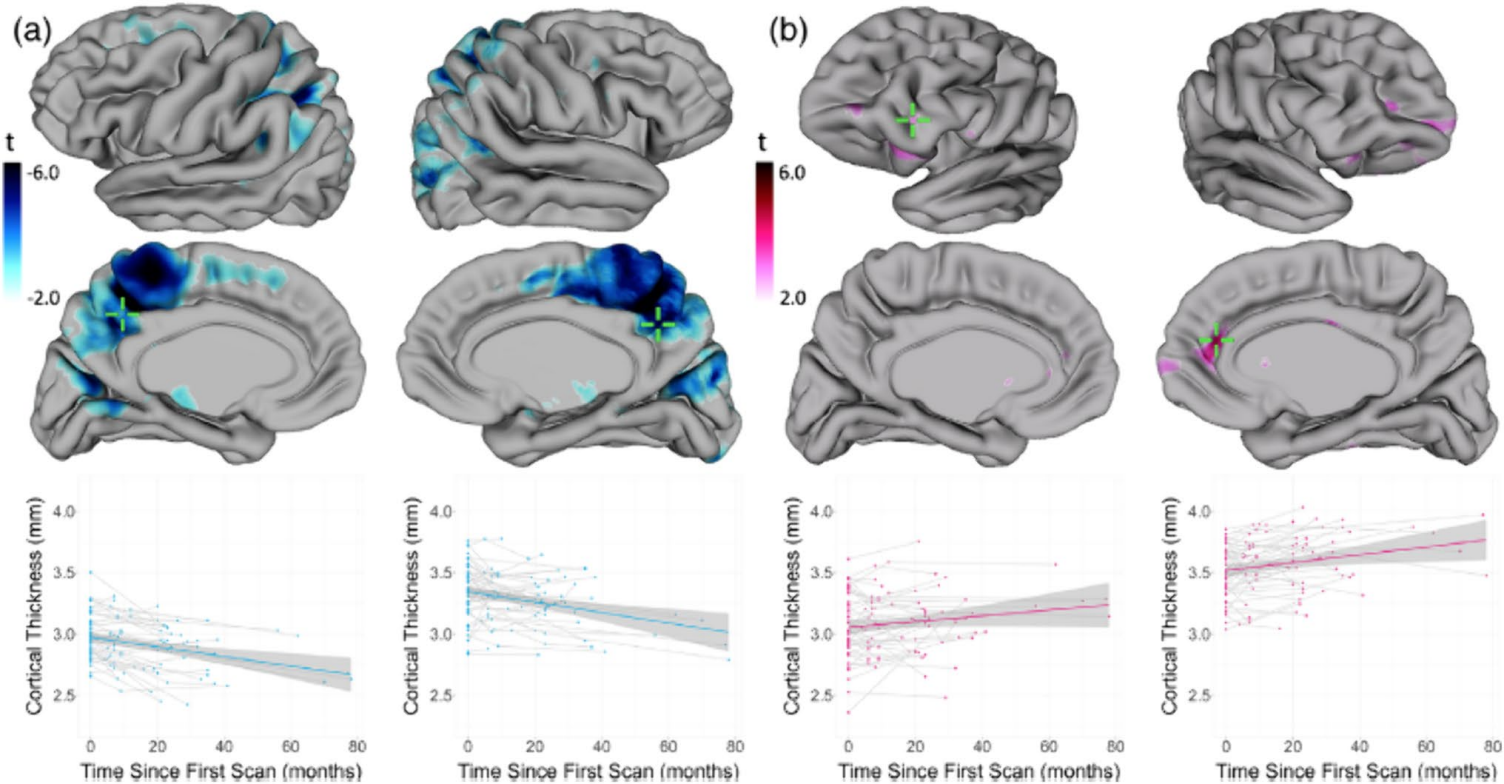
Effect of time on cortical thickness in TBI group. Coloured areas represent the location of vertices that demonstrate a significant change in cortical thickness over time (10% false discovery rate [FDR] corrected), where darker shades reflect the most substantial changes (see colour bar). Cortical thickness at single vertices indicated by crosshairs on the cortical surfaces above are plotted to illustrate cortical thickness changes over time. **a** Regions of the cortex decrease in thickness over time. **b** Regions of the cortex increase in thickness over time (Mazaharally et al. 2022)

The changes in the brain following a TBI are complex and have been investigated using various radiographical studies over the recent years, this includes investigating the impact that TBI may have on brain function using MRI, functional MRI (fMRI) and Angio-CT (Ramos-Cejudo et al. 2018). Mazaharally et al. (2022) investigated cortical changes in the brain following TBI using MRI scans from 67 individuals with clinically confirmed TBI taken at 4-time points post-TBI ranging from 5 months to 7 years, compared with 18 controls whose scans were taken at 2-time points. Their study found that the post-TBI individuals showed increased cortical thickness in the frontal regions however cortical thickness reductions in the posterior cingulate/precuneus regions which can be seen in Fig. 1 (Mazaharally et al. 2022). This shows regional differences in response to TBI, and further investigation into this topic is required alongside histological studies to investigate the role that astrocytes may play in changing the cortical morphometry post-TBI.

**Fig. 2:**
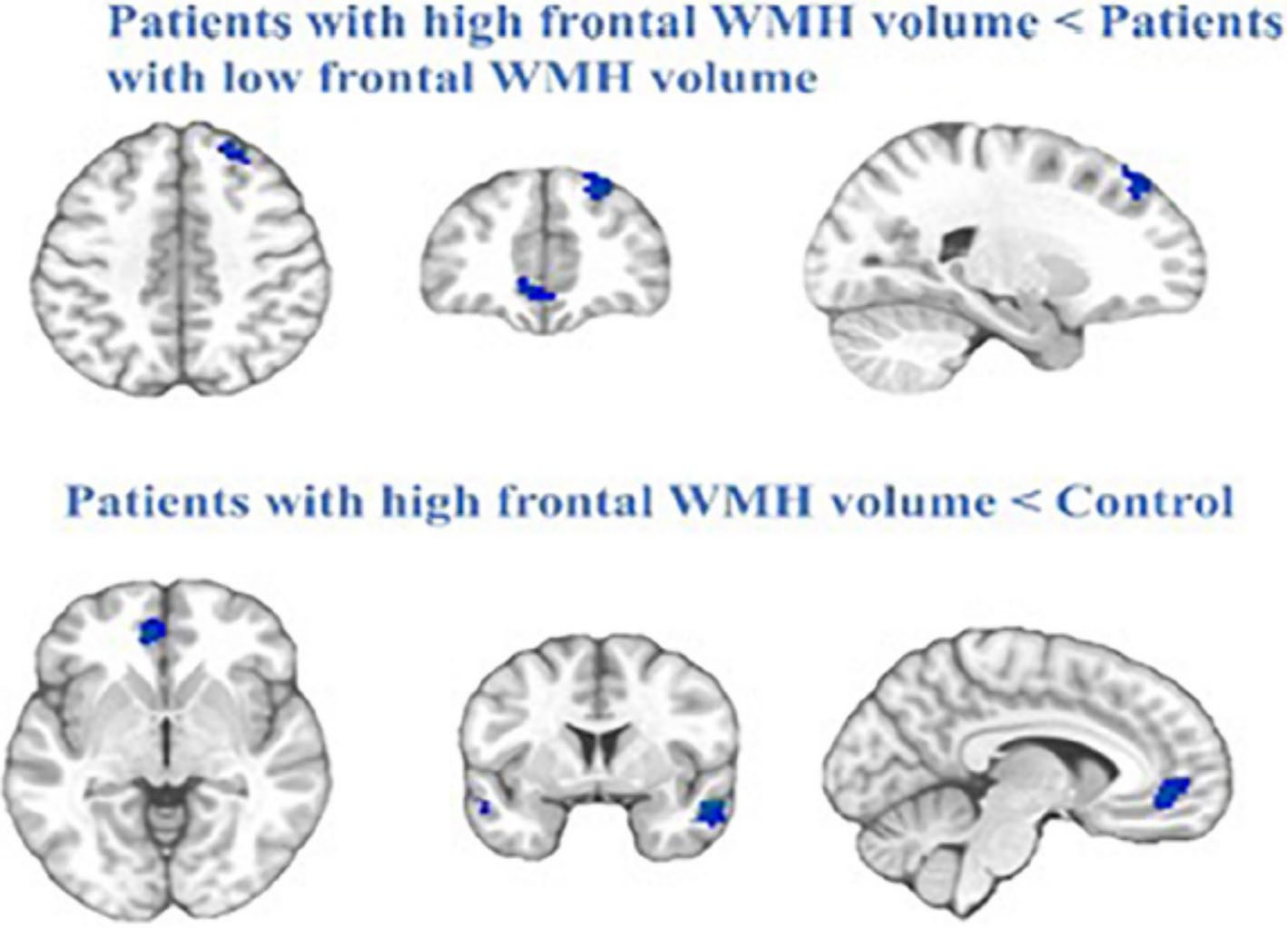
The blue represents regions of reduced resting-stated functional connectivity in the patients with high frontal WMH volume (Patient-A) compared with patients with low frontal WMH volume (Patient-B) and the control. Cluster extent threshold of *P *value of 0.01 using an Family-Wise Error (FWE) correction for multiple comparisons (Zhang et al. 2022)

Another study used MRI and fMRI on a sample of 46 patients who suffered mTBI matched with 46 healthy controls to investigate changes in white-matter neurons which may be caused by TBI (Zhang et al. 2022). Their study found increased WMH volume in the frontal and parietal lobes, which correlated with poorer information processing speed via tests such as the digital symbol coding test and trail-making test (Zhang et al. 2022). Similarly, white WMHs in the frontal lobe were associated with significantly lower functional connectivity in areas such as the right middle temporal gyrus, left middle frontal gyrus, left anterior cingulate cortex, and the right superior frontal gyrus (Zhang et al. 2022). The researchers postulated that the frontal white-matter damage, impaired the functional connectivity of the default mode network as seen in Fig. 2, resulting in poorer information processing speed and cognitive impairment (Zhang et al. 2022).

Researchers have used fMRI to compare brain patterns and cognitive impairments between three groups, one mTBI group with 33 patients, one AD group with 66 patients, and healthy controls with 81 patients (Rostowsky and Irimia 2021). Their study found that the degree of acute cognitive impairment 48 h post-injury (measured using the Montréal Cognitive Assessment), was correlated with the severity of brain atrophy patterns found in AD (Rostowsky and Irimia 2021). Contrarily, a recent article investigated the long-term impact of TBI with loss of consciousness, and while an association was found between TBI with loss of consciousness and lower white-matter volumes, no association was found between TBI history and white-matter WMHs and other brain volumes (Grasset et al. 2023).

Other studies have investigated the relationship between young adults having a TBI and hyperactive/impulsive behaviours (Cao et al. 2022). In their study, 89 participants aged between 18 to 26 years were included, 44 participants had suffered from TBI and 45 were group-matched healthy controls (Cao et al. 2022). The participants who had suffered TBI showed significantly altered neurite densities in the bilateral parietal and frontal lobes of the grey matter, significantly decreased neurite densities in the left precentral gyrus and superior longitudinal fasciculus, alongside changes in the white matter which is connected to these areas (Cao et al. 2022). Their study also found a strong relationship between decreases in neurite densities in the left precentral gyrus and superior longitudinal fasciculus and hyperactive/impulsive behaviours, as measured by Conner’s Adult ADHD Self-Reporting Scale (Cao et al. 2022).

MRI and Angio-CT have been utilised to view the cerebrovascular impact of TBI. One study found that a variety of cerebrovascular impairments following TBI such as post-traumatic cerebral venous thrombosis, lateral and sigmoid sinus impairments and thrombosis in the jugular vein (Chtara et al. 2023), while another study found TBI commonly leads to post-traumatic pulmonary embolism, cerebral contusions, and subdural/extradural haemorrhages (Haddad et al. 2022). Some post-TBI studies have implicated cerebrovascular injuries as having a role in expediting AD pathology (Ramos-Cejudo et al. 2018). This pathology is suggested to increase due to endothelial cells and blood-–brain barrier damage which may inhibit the clearance of waste in the brain and increase tau pathology, alongside cerebrovascular inflammation which may cause neurotoxic glial activation such as neurotoxic astrocytes (Ramos-Cejudo et al. 2018). The immune response, initiated by reactive astrocytes following cerebrovascular inflammation plays an important role in the neurodegeneration which may result from TBI, and these different areas should all be investigated due to the potential therapeutic benefits that may be gained (Zhou et al. 2020).

## Conclusion

Whilst a variety of studies have investigated TBI and neurodegeneration, whether a single mTBI results in an increased risk of AD is still in debate, a variety of studies suggest that other factors may be at play such as loss of consciousness at the time of TBI, and cognitive impairment following a TBI. The microglial and astrocyte changes in the brain during AD and following TBI are of increasing interest in recent years, and while studies have investigated the impact of neurotoxic astrocytes post-TBI by analysing C3 in post-TBI mice, no studies yet have investigated C3 in post-mortem human samples. Similarly, the exact distribution of neurotoxic and neuroprotective astrocytes following TBI is unknown. Due to the variety of proteins released by neurotoxic and neuroprotective astrocytes, further studies investigating the variety of gene expressions may assist in discovering the roles of neurotoxic and neuroprotective astrocytes post-TBI, alongside seeing the distributions of these reactive astrocytes in different portions of the brain. This could gain insight into the immunological changes in the brain that occur as a result of TBI. Furthermore, additional studies could explore potential sub-groups of the neurotoxic and neuroprotective reactive astrocytes, with different upregulation and downregulation patterns. This review may provide insight into the functions of neurotoxic and neuroprotective astrocytes following TBI, and in the future may build upon the knowledge that results in improved outcomes for patients with short-term and long-term complications following TBI.
